# Lead Induces Apoptosis and Histone Hyperacetylation in Rat Cardiovascular Tissues

**DOI:** 10.1371/journal.pone.0129091

**Published:** 2015-06-15

**Authors:** Li-Hui Xu, Fang-Fang Mu, Jian-Hong Zhao, Qiang He, Cui-Li Cao, Hui Yang, Qi Liu, Xue-Hui Liu, Su-Ju Sun

**Affiliations:** 1 Department of Biochemistry and Molecular Biology, Hebei Medical University, Shijiazhuang, 050017, China; 2 College of Public Health, Hebei Medical University, Shijiazhuang, 050017, China; 3 The Second Hospital, Hebei Medical University, Shijiazhuang, 050000, China; 4 Laboratory of Neurobiology, Institute of Basic Medicine, Hebei Medical University, Shijiazhuang, 050017, China; Max-Delbrück Center for Molecular Medicine (MDC), GERMANY

## Abstract

Acute and chronic lead (Pb) exposure might cause hypertension and cardiovascular diseases. The purpose of this study was to evaluate the effects of early acute exposure to Pb on the cellular morphology, apoptosis, and proliferation in rats and to elucidate the early mechanisms involved in the development of Pb-induced hypertension. Very young Sprague-Dawley rats were allowed to drink 1% Pb acetate for 12 and 40 days. Western blot analysis indicated that the expression of proliferating cell nuclear antigen (PCNA) decreased in the tissues of the abdominal and thoracic aortas and increased in the cardiac tissue after 12 and 40 days of Pb exposure, respectively. Bax was upregulated and Bcl-2 was downregulated in vascular and cardiac tissues after 40 days of Pb exposure. In addition, an increase in caspase-3 activity was observed after 40 days of exposure to Pb. In terms of morphology, we found that the internal elastic lamina (IEL) of aorta lost the original curve and the diameter of cardiac cell was enlarged after 40 days. Furthermore, the exposure led to a marked increase in acetylated histone H3 levels in the aortas and cardiac tissue after 12 and 40 days, than that in the control group. These findings indicate that Pb might increase the level of histone acetylation and induce apoptosis in vascular and cardiac tissues. However, the mechanism involved need to be further investigated.

## Introduction

Lead (Pb) is a common environmental and industrial pollutant with no beneficial biological role. The metal is ubiquitous in the environment and toxic to humans. The persistence of Pb in the blood of animals and humans, and the associated health risk are a topic of current debate and concern [1,2].

Recent research has focused on understanding its toxic effects on the developing peripheral and central nervous system [3,4,5]. However, Pb also impacts other organs and systems, including the hematologic and skeletal systems [6,7] and those involved in cardiovascular diseases [8, 9].

Although the cardiovascular system is not typically considered a target of Pb toxicity, but Pb is toxic to both the heart and vascular smooth muscles. A number of animal studies and epidemiologic investigations suggest that Pb may elevate blood pressure [10,11,12,13]. Furthermore, a significant association between Pb levels in the blood and hypertension prevalence was found among U.S. women aged 40 to 59 years [8]. Several mechanisms by which Pb exposure causes hypertension and cardiovascular disease have been proposed. Gonick et al. demonstrated that Pb exposure could induce vascular oxidative stress [14]. Other researchers showed that Pb exposure could reduce the level of nitric oxide and increase the level of endothelin and inhibit the activity of Na^+^, K^+^-ATPase [15,16,17,18]. Furthermore, Pb might also disturb Ca^2+^-dependent signaling pathways and nuclear factor erythroid 2-related factor (Nrf2) pathways [19].

Apoptosis and proliferation play an important role in the vessel wall in atherosclerosis and arterial remodeling. It means that cell death and cell proliferation may result in changes in the vascular architecture during development and disease [20]. Epigenetics investigates heritable changes in gene expression occurring without changes in DNA sequences. Recent investigations have identified a number of environmental toxicants that cause altered methylation and acetylation of human repetitive elements or genes [21, 22]. The acetylation or deacetylation of histone N-terminal tails alter the interaction between histones and DNA molecules in chromatin [23], and this remodeling of chromatin has been identified as a key step in the regulation of gene expression [24]. In general, hyperacetylation is associated with transcriptional activation, whereas hypoacetylation is associated with repression. Histone acetylation has been demonstrated to contribute to proliferation arrest, differentiation, and/or apoptotic cell death of transformed cells *in vitro* and *in vivo* [25,26]. However, fewer studies reported the epigenetic alterations for vascular tissue acutely exposed to Pb.

In this study, we developed an experimental model of acute Pb exposure in rats [12]. We then analyzed the effects of this treatment on: 1) the aortic and cardiac structure; 2) cell proliferation and apoptosis in the cardiovascular; 3) histone acetylation that occurs in response to early Pb exposure. The study aimed to elucidate the mechanisms involved in the very early development of Pb-induced vascular toxicity.

## Materials and Methods

### Animals and treatment

The current study was performed in accordance with the Guide for the Care and Use of Laboratory Animals of the National Institutes of Health. The animal experimental protocols were approved by the Institutional Animal Care and Use Committee of Hebei Medical University.

Sixty Sprague–Dawley rats were obtained at 4–5 weeks of age (weighing 99–114g) from the Laboratory Animal Center in Hebei Medical University. The rats were allowed to acclimatize for two weeks to the initiation of the study and were assigned into four general groups composed of 15 rats. Two groups were fed with regular rat chow and distilled water supplemented with 1% Pb acetate for 12 and 40 days, respectively. Two control groups were provided with regular rat chow and distilled water without any treatment throughout the observation period. The concentration of Pb^2+^ chosen in this study as 1% Pb acetate was based on the study reported by Bagchi et al. [12]. At the end of study, rats were fasted overnight and euthanized by phlebotomy through celiac aorta puncture. Blood was collected to measure the concentration of Pb. The abdominal and thoracic aortas and hearts were harvested, cleaned in icy phosphate balanced solution. The abdominal and thoracic aorta and cardiac tissue of 5 rats in each group (n = 5) was harvested and fixed in 10% phosphate balanced solution-buffered formaldehyde. The aorta and cardiac tissue from the remaining rats in each group were frozen in liquid nitrogen and then stored at −80°C until processing for western blot.

### Histomorphological examination

To assess the structural changes in the aorta and heart of the rats (exposed to Pb for a short-term period), after fixation in 10% PBS-buffered formaldehyde, processing, and embedding in paraffin, six serial sections (5 μm thick) were obtained that were sectioned at equally spaced intervals, and stained with hematoxylin and eosin (H&E) staining for morphometry.

### Western blotting

The heart and abdominal and thoracic aortas were homogenized with RIPA lysis buffer containing protease inhibitors. The lysate was centrifuged at 12,000 × *g* and the insoluble material was removed. The protein concentration of the soluble material was determined via the Bradford Kit (Invitrogen, Carlsbad, CA, USA). Aliquots of protein extract (80 μg) were separated by 10% SDS-PAGE for PCNA, Bax, and Bcl-2 subunit. The proteins were transferred to a polyvinylidene fluoride membrane (Schleider and Schuell, Dassel, Germany). The blots were probed overnight at 4°C with the following rabbit anti-rat polyclonal antibodies: PCNA (1:400, Sigma-Aldrich, St Louis, MO, USA), Bax (1:400, Cell Signaling Technology, Danvers, MA, USA), Bcl-2 (1:400, Santa Cruz Biotechnology, Santa Cruz, CA, USA). Membranes were incubated with anti-rat (1:5000, Sigma-Aldrich) immunoglobulin antibody conjugated to horseradish peroxidase. After being washed thoroughly, immunocomplexes were detected using an enhanced horseradish peroxidase/luminal chemiluminescence system (ECL Plus, Amersham International, Little Chalfont, UK) and film (Hyperfilm ECL International). Signals on the immunoblot were quantified using the National Institutes of Health Image V1.56 computer program. The same membrane was used to determine β-actin expression using a mouse monoclonal antibody for β-actin (1:5000, Sigma-Aldrich), and after being washed, it was incubated with an anti-mouse (1:5000, Sigma-Aldrich). Nuclear extracts were isolated from the heart and abdominal and thoracic aortas for histone acetylation (H3K9). The H3K9 antibody was a rabbit anti-rat polyclonal antibody (1:500, Cell Signaling Technology). Other reagents for western blotting were purchased from Sigma-Aldrich.

### Caspase-3 activity assay

The activity of caspase-3 was determined using the Caspase-3 activity kit (Beyotime Institute of Biotechnology, Haimen, China) following the manufacturer’s protocol. To evaluate the activity of caspase-3, the tissue lysates were prepared after various designated treatments. Assays were performed on 96-well microtiter plates by incubating 10 μL protein from the tissue lysate per sample in 80 μL reaction buffer (1% NP-40, 20 mM Tris–HCl (pH 7.5), 137 mM Nad, and 10% glycerol) containing 10 μL caspase-3 substrate (Ac-DEVD-pNA) (2 mM). Lysates were incubated at 37°C for 4 h. Samples were measured by using a microplate reader at an absorbance of 405 nm.

### Whole blood Pb content

Pb content of the whole blood was determined using a Graphite furnace atomic absorption spectrometric TAS 990 (Beijing, China). Hollow cathode lamp of Pb was used at wavelength of 283.3 nm. The recovery of Pb that had been added externally to control samples was found to be consistently greater than 96%. Whole-blood Pb values were expressed as micrograms per liter.

### Blood pressure (BP)

Rats were preheated in a chamber at 35°C for 10 min and then placed in plastic restrainers. A cuff with a pneumatic pulse sensor was attached to the tail. Rats were allowed to get habituated to this procedure for 7 d before actual experiments were performed. BP values were recorded on a Model BP-600 (Chendu Taimeng Co., Ltd., Chengdu, China) with heating and were averaged from at least three consecutive readings obtained from each rat.

### Data presentation and analysis

All data are expressed as means ± SD. Unpaired t-tests were performed and a p value <0.05 was considered to be statistically significant.

## Results

### Blood Pb level

Firstly, we assessed the level of blood Pb. On day 12, the average blood Pb level of the control group was 68.2 μg/L, whereas in the Pb exposure group, it was 193.3 μg/L. On day 40 of Pb exposure, the levels of blood Pb were 69.1 μg/L and 245.9 μg/L in the control and Pb exposure group, respectively (S1 Table). These differences were statistically significant.

### Blood pressure

**Fig 1:**
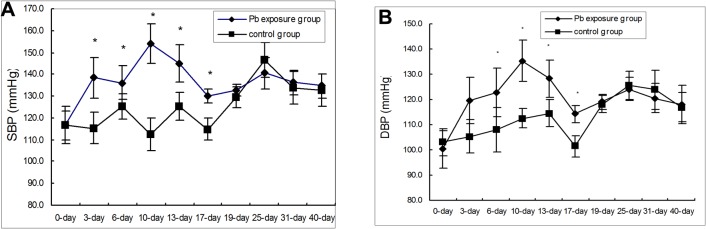
Effect of Pb exposure on blood pressure. A. Effects of Pb exposure on systolic blood pressure. B. Effects of Pb exposure on diastolic blood pressure. n = 6 Data are the means ± SD. *p < 0.05 compared with the control group

Between the 6^th^ and 17^th^ day, the systolic blood pressure (SBP) (Fig 1A, S1A Data and S1B Data) and diastolic blood pressure (DBP) (Fig 1B, S1C Data and S1D Data) of the Pb-exposed rats increased significantly over that of the control rats. Following this brief period, the SBP and DBP of the Pb-exposed rats return to levels similar to that of controls. After the 19^th^ day of Pb exposure, the average SBP and DBP of the two groups were similar.

### Pathological morphology of vascular tissue and heart

**Fig 2:**
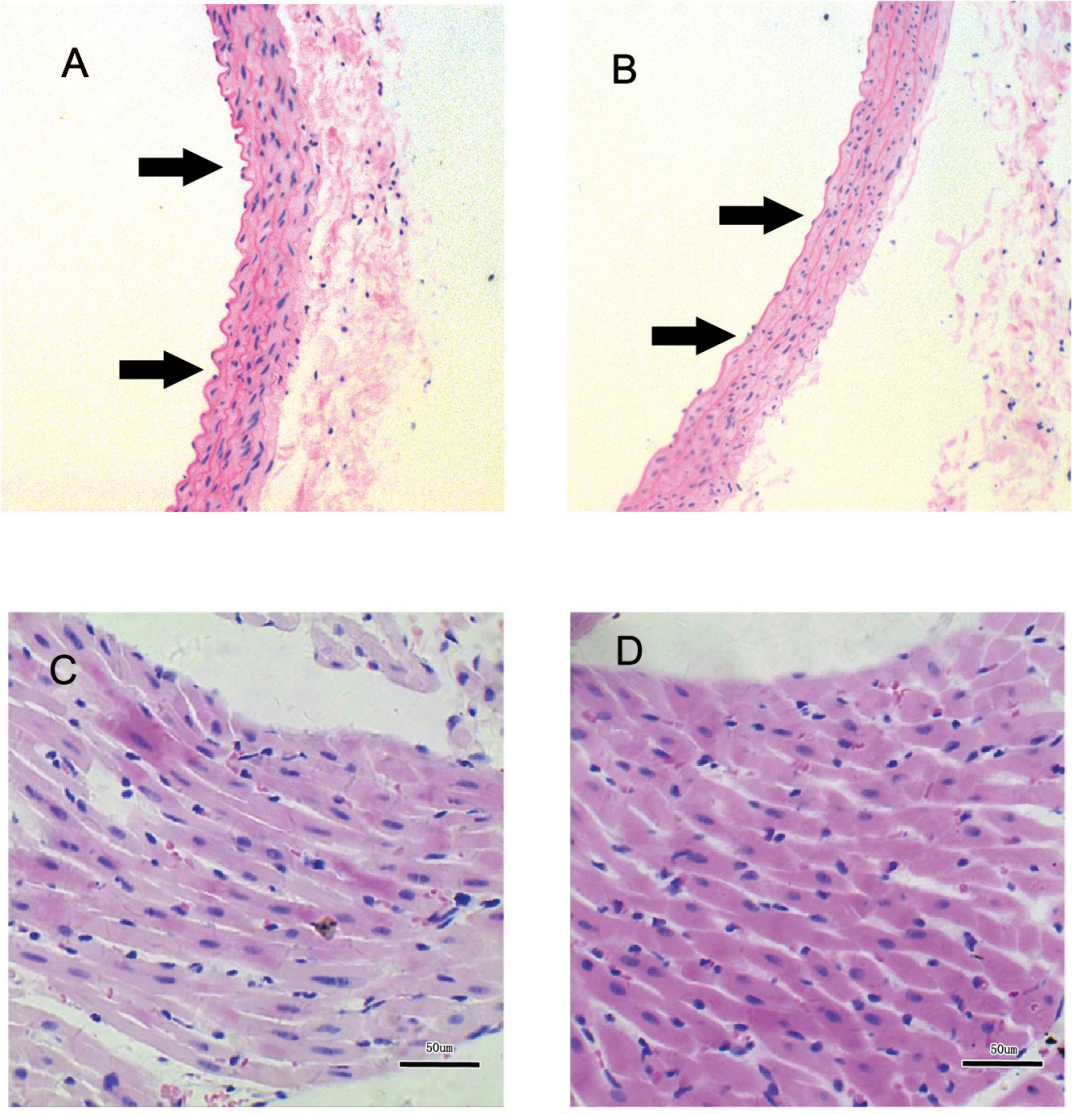
The histological structure of SD rat aorta and cardiac tissue by H&E staining method at day 40 after Pb exposure. A is the aorta tissue of control rat and B is the aorta tissue of Pb exposed rat (100×). C is the cardiac tissue of control rat and D is the cardiac tissue of Pb exposed rat (400×).

Light microscopic analysis of vascular and heart tissues of rats from the Pb exposure and control group is shown in Fig 2. In the light microscopy examination, the internal elastic lamina (IEL) of aorta in the 40-day Pb exposure group become a straight line and lost its original curve. However, the IEL in the control group kept the original curve (Fig 2A and 2B). For the cardiac tissue, the cardiac cells in 40-day Pb-exposed rats presented a significantly larger diameter than those of the control rats (Fig 2C and 2D).

### Pb effect on the proliferation of the aortas and heart

**Fig 3:**
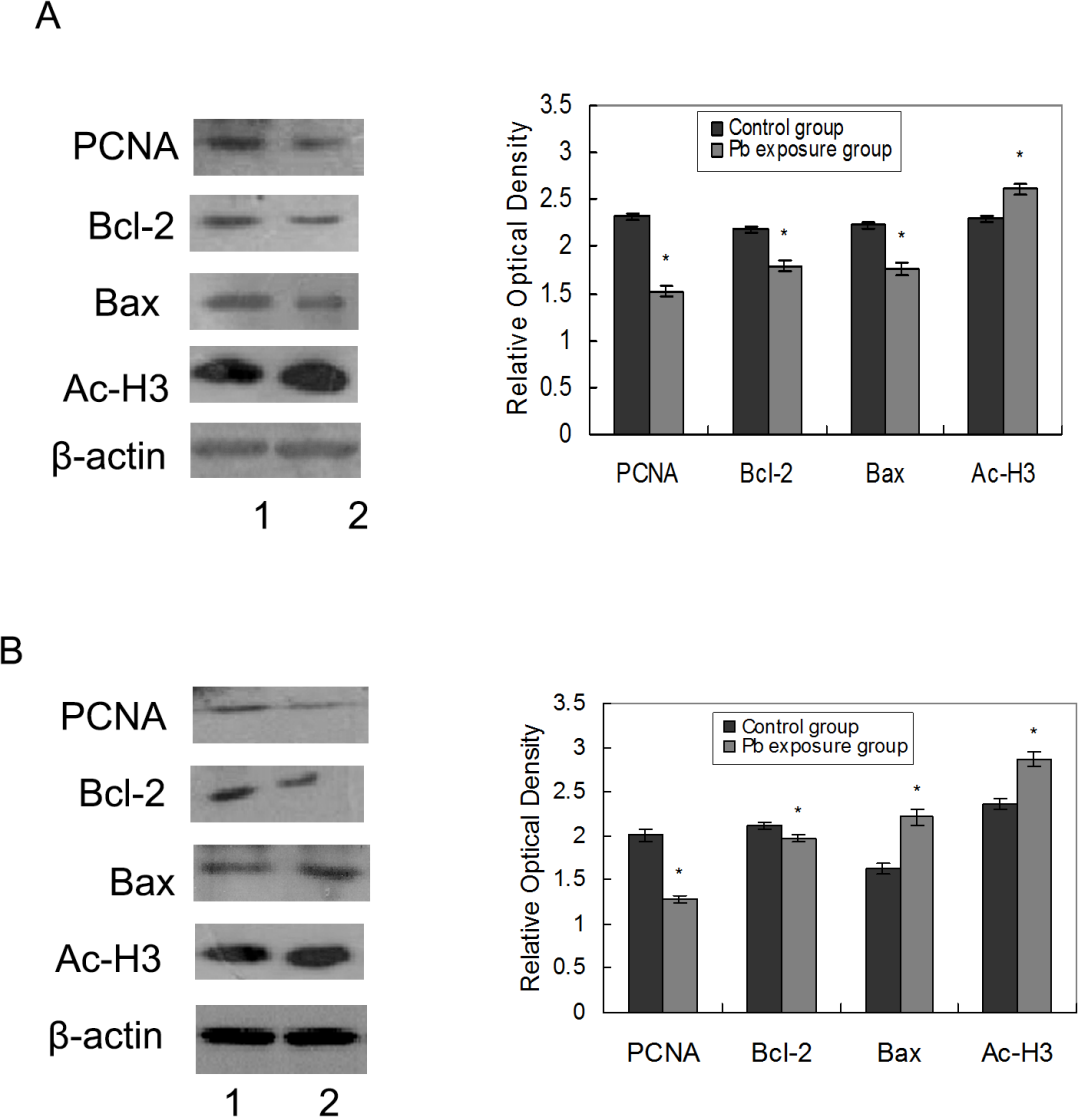
Effects of Pb on vascular tissue protein expression. Effects of Pb exposure on the protein expression of PCNA, Bcl-2, Bax, and acetyl-H_3_ in abdominal and thoracic aorta tissue at 12 days after Pb exposure (A) and 40 days after Pb exposure (B). The corresponding bar graphs represent densitometric quantification. n = 6 experiments. Probability values are indicated above bars: *p < 0.05 versus control. 1 stand for control group, 2 stand for Pb exposure group.

**Fig 4:**
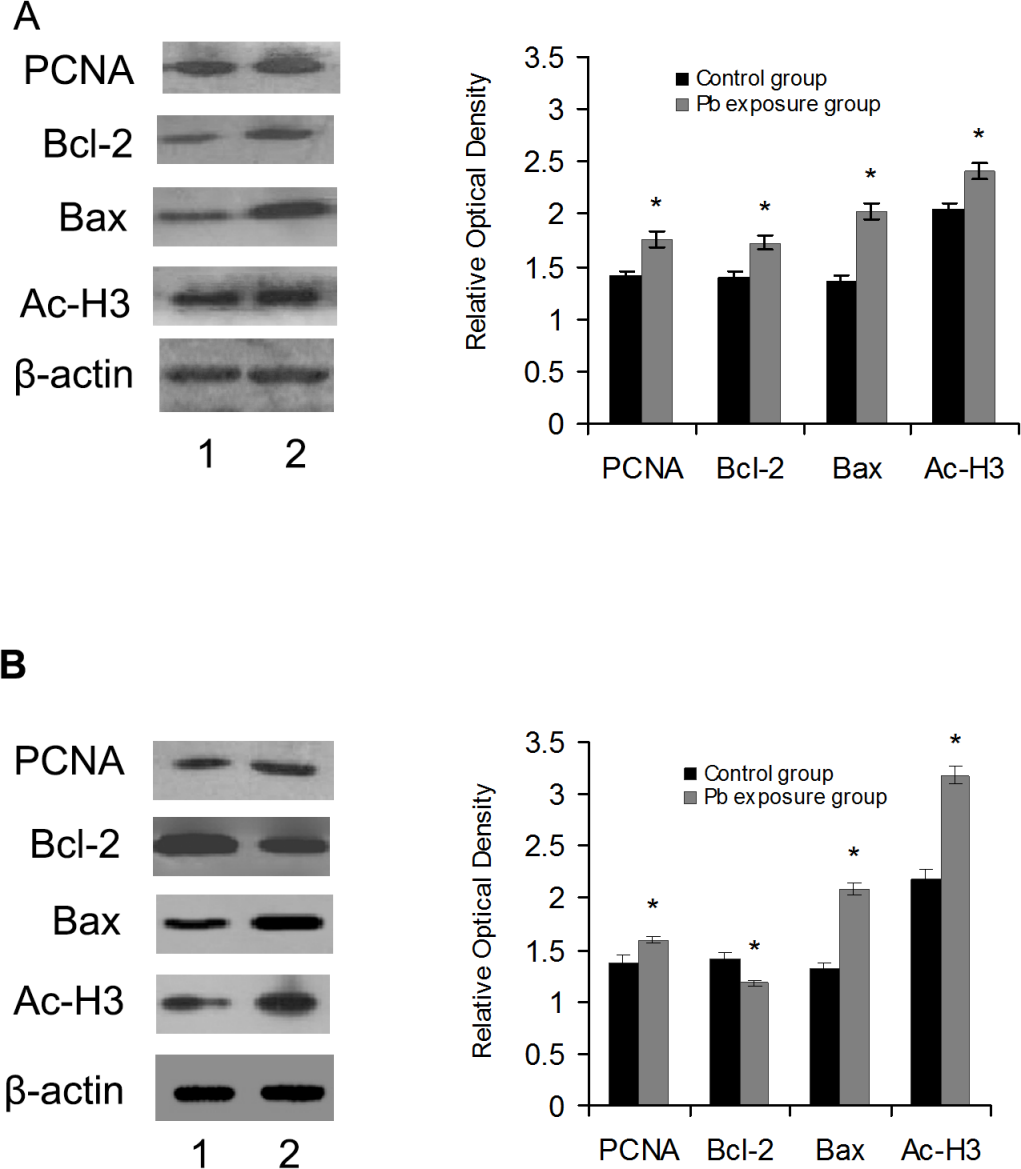
Effects of Pb on cardiac tissue protein expression. Effects of Pb exposure on the protein expression of PCNA, Bcl-2, Bax and acetyl-H_3_ in cardiac tissue at 12 day after Pb exposure (A) and 40 day after Pb exposure (B). The corresponding bar graphs represent densitometric quantification. n = 6 experiments. Probability values are indicated above bars: *p < 0.05 versus control. 1 stand for control group, 2 stand for Pb exposure group.

The effects of Pb on the proliferation of the abdominal and thoracic aortas and cardiac tissues were examined by analyzing PCNA expression by western blot. As shown in Fig 3, PCNA expression decreased in aortas both at day 12 or and day 40 after Pb exposure. In contrast, PCNA levels increased in cardiac tissues on both experimental days (Fig 4).

### Pb modulated apoptosis-related factor expression in the aortas and hearts

We evaluated whether Pb induced apoptosis through the activation of Bax and inhibition of Bcl-2 by western blot analysis. Fig 3 shows that Pb exposure affected the levels of the apoptosis-related factors in the aortas. The relative optical density of the proapoptotic protein, Bax, was lower in the 12-day Pb exposure group than that in the control group. In the 40-day Pb-exposed rats, the expression level of Bax was increased. However, the expression of the anti-apoptotic protein Bcl-2 was lower in the 12 and 40-day Pb-exposed rats. After 12 days of exposure, the ratios of Bcl-2 to Bax of the control and Pb exposure group were 0.98 and 1.01 (Table 1), respectively. After 40 days of exposure, the ratios of Bcl-2 to Bax were 1.30 and 0.89 (Table 1) for the control and Pb exposure group, respectively.

**Table 1:** The ratio of Bcl-2/Bax in vascular tissue in two groups.

Group	n	12-day	40-day
control group	6	0.98 ± 0.08	1.30 ± 0.05
Pb exposure group	6	1.01 ± 0.07	0.89 ± 0.06*

*P < 0.05

The apoptosis-related factors in the cardiac tissue are presented in Fig 4. Compared to the control group, Bax increased in the cardiac tissues in the 12 and 40-day Pb exposure group. In comparison to the control group, after 12 days of exposure, Bcl-2 increased, whereas, after 40 days of exposure, Bcl-2 decreased compared to that of the control group.

At 12 days of Pb exposure, the ratios of Bcl-2 to Bax of the control and Pb exposure group were 1.03 and 0.93 (Table 2), respectively. At 40 days of Pb exposure, the ratios of Bcl-2 to Bax were 1.08 and 0.56 (Table 2) in the control and Pb exposure group, respectively.

**Table 2:** The ratio of Bcl-2/Bax in cardiac tissue in two groups.

Group	n	12-day	40-day
control group	6	1.03 ± 0.07	1.08 ± 0.05
Pb exposure group	6	0.93 ± 0.07	0.56 ± 0.04*

*P < 0.05

### Activation of caspase 3 in cardiovascular exposed to Pb

**Fig 5:**
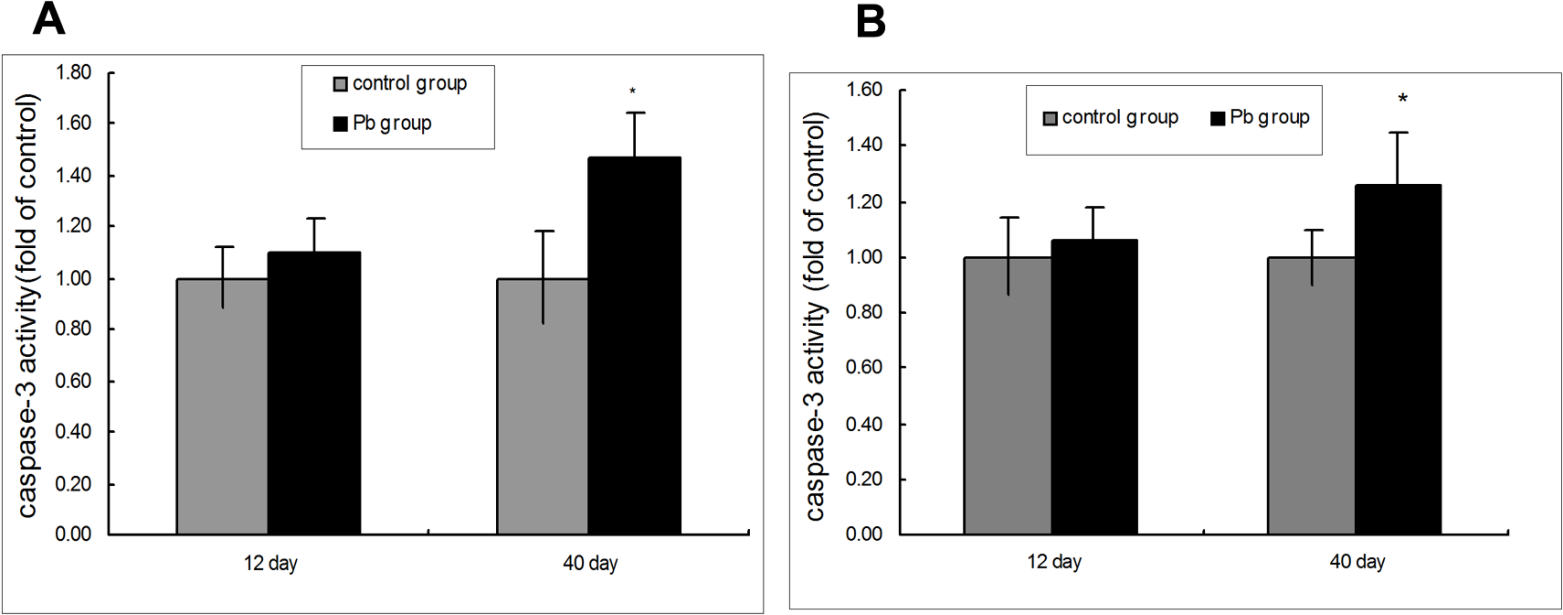
Pb-induced caspase-3 activation in cardiovascular tissue. (A) Caspase-3 activity in rat vascular following Pb exposure. (B) Caspase-3 activity in rat cardiac tissue following Pb exposure. The rats were treated with 1% Pb acetate for 12 and 40 days. n = 6. Data are the means ± SD. *p < 0.05 compared with the control group. The relative activities of caspase-3 shown are calculated from the average of experiment. Each value was expressed as the ratio of caspase-3 activation level to the control level, and the value of the control was set to 1.

Since caspase-3 has been shown to play a pivotal role in the execution phase of apoptosis induced by diverse stimuli [27], we measured caspase-3 activity. Caspase-3 activation, analyzed by measuring the levels of p-nitroanilide cleaved from the substrate N-Ac-DEVD-pNA. There were no significant difference between the 12-day Pb exposure group and the control group (Fig 5 and S2A Data). Rats treated with Pb for 40 days showed significant increases in caspase-3 activity (Fig 5 and S2B Data). These results implicated that Pb-induced apoptosis occurs through the activation of common executors of apoptosis such as caspase-3.

### Effect of Pb on Acetylation of Histone H3 in vascular and heart tissue

As core histones are mainly acetylated on specific lysine residues, the specific lysine acetylation of core histones H3 was analyzed. We examined the effects of Pb on the acetylation of histone H3 at Lys9 in the cardiovascular by western blot using an acetylated histone type specific antibody. As shown in Fig 3 and Fig 4, Pb exposure for 12 and 40 days led to marked increases in acetylated histone H3 levels in the abdominal and thoracic aortas and cardiac tissues.

## Discussion

There is a growing awareness of the pleiotropic effects of Pb, with targets of toxicity including multiple tissues such as the cardiovascular system. The National Health and Nutrition Examination Surveys (NHANES III) demonstrated that Pb exposure increases SBP and DBP [8]. Many studies reported that Pb^2+^ was able to induce hypertension and arteriosclerosis in animals and humans [9, 11, 12, 19]. However, the mechanism involved in the development of atherosclerosis and vascular diseases is still not clear. In this study, we aimed at investigating the short-term effect of Pb on the proliferation, apoptosis, and histone acetylation of cardiovascular tissues.

Apoptosis and proliferation appear to play a significant role in many vascular or vascular-related diseases such as atherosclerosis and hypertensive vascular diseases. It has been shown that apoptotic death of vascular smooth muscle cells is an important component, especially in the early phase of vascular injury [28]. PCNA was originally characterized as a DNA sliding clamp for replicative DNA polymerases and as an essential component of the eukaryotic chromosomal DNA replisome. The BCL-2 family of proteins includes both pro- as well as anti-apoptotic molecules. One characteristic of the members of this family is to form homo- as well as heterodimers, suggesting neutralizing competition between these proteins. The ratio between these two subsets could help identify, in part, the susceptibility of cells to a death signal [29]. Cysteine aspartases (caspases), a protease family, are known to be required for apoptosis induced by various stimuli [30]. Among mammalian caspases, comprising at least 14 known members, caspase-3 is thought to be the main effector of caspases and has been identified as being activated in response to cytotoxic drugs [30]. Caspase-3 activation is an important step in the execution phase of apoptosis. Our results showed that Pb could induce the downregulation of Bcl-2 and Bax in vascular tissue and the upregulation Bcl-2 and Bax in cardiac tissues of rats after 12 days of exposure. The ratio of Bcl-2/Bax did not change and the activity of caspase 3 did not significantly increase. This result indicates that the pro- as well as anti-apoptotic molecules/proteins still maintain a regulated balance in vascular and cardiac cells that allows programmed cell death to occur in response to Pb damage. After Pb exposure for 40 days, the expression of Bax in vascular and cardiac tissues increased, while Bcl-2 expression was lower. This means that the balance was deregulated by the overexpression of pro-apoptotic factors. Our results indicate that Pb could upregulate Bax expression and inhibit Bcl-2 expression. Meanwhile, caspase 3 activity increased. Therefore, Pb promotes vascular and cardiac cell apoptosis. Carsia et al. [31] reported that Pb causes growth arrest in cultured rat aorta smooth muscle cells at a high concentration (500 μM). In contrast, a low concentration of Pb citrate (100 μM) causes hyperplasia in cultured rat aorta smooth muscle cells [31]. Fujiwara [32] reported that Pb has been shown to stimulate proliferation of bovine aortic smooth muscle cells in a concentration-dependent manner (0.5–10μM). Additionally, one human study on intimal and medial thickness (IMT) in healthy young women suggested that increased serum Pb levels are associated with an increased risk for high IMT, suggesting a role for Pb in the development of intimal (plus medial) hyperplasia [19]. Thus, exposure to different concentrations of Pb may affect the proliferation of vascular tissue. The present study showed that Pb exposure could inhibit proliferation and promote apoptosis of vascular tissue. Meanwhile, Pb exposure could damage the IEL of aorta tissue. Either Pb-induced vascular tissue apoptosis or the early high pressure might result in IEL damage. The IEL is a layer of elastic tissue that forms the outermost part of the tunica intima of blood vessels. It represents a flexible barrier between the endothelium and inner smooth muscle cell layer [33]. The injured IEL might influence the modulation of diffusion across the artery wall.

Few studies showed the effect of Pb exposure on cardiac tissue. Our results showed that Pb could promote both cardiac cell apoptosis and proliferation. The pathological results showed the cardiac cells in Pb exposed rats presented a significantly larger diameter than those of the control rats. The hypertrophy of cardiac muscle cells might be due to the early high blood pressure, endocrine disturbance, and Pb toxicity, resulting in apoptosis of the cardiomyocytes. Apoptosis causes the compensatory hypertrophic growth of individual cardiomyocytes, resulting in an increase in PCNA expression.

In order to further elucidate the molecular mechanism responsible for the Pb-induced apoptosis in cardiovascular tissue, the effect of Pb on histone acetylation was analyzed. Studies suggest that the level of histone acetylation can affect cell growth [25,26,34]. Many studies reported that histone deacetylases (HDAC) inhibitors (HDAC inhibitors induce histone hyperacetylation) could alter the expression of the Bcl-2 family protein [30,31]. Alterations in Bcl-2 protein expression include decrease in Bcl-2, Mcl-1, Bcl-XL, and XIAP expression, or increase in Bax and Bak expression [35,36,37,38,39,40, 41]. The HDAC inhibitor FR901228 could downregulate the expression of Bcl-2 and Bcl-XL, and activate caspase-9 and caspase-3 in small cell lung cancer cell lines [38]. Another study found that the apoptosis induced by suberic bishydroxamate is associated with conformational changes in Bax and with changes in mitochondrial membrane permeability and caspase-3 activation [37]. The HDAC inhibitors, TAS and SK-7041, could activate caspase-3 and inhibited the expression of Mcl-1 and Bcl-XL, but did not affect the expression of Bcl-2, Bax, or Bak [40].Our result showed that histone acetylation status increased in cardiac and aorta tissue after 12 and 40 days of Pb exposure in rats. Pb-induced histone hyperacetylation could result either from a stimulation of histone acetylase activity or an inhibition of HDAC activity. Whether and how the histone hyperacetylation inhibited the expression of Bcl-2 and increased the expression of Bax need to be further investigated.

## Conclusions

Our study demonstrated that Pb inhibited the proliferation of vascular tissue by reducing the expression of PCNA and enhanced apoptosis by increasing Bax level and decreasing Bcl-2 level in the early Pb exposure phase. In addition, an increase in caspase-3 activity was observed after 40 days of Pb treatment. Meanwhile, Pb exposure could damage the aorta IEL. The vascular tissue apoptosis might result in IEL damage. Meanwhile, Pb could promote cardiac cell apoptosis and proliferation of the cardiac tissue and cause cardiac cell hypertrophy. Furthermore, Pb could enhance the level of histone acetylation. Whether Pb promoted apoptosis of vascular and cardiac tissue through induction of apoptosis mediated by the activation of histone hyperacetylation needs to be further explored.

## Supporting Information

S1 Data Blood pressure values of all samples. A. Systolic Blood Pressure values of Pb exposure group. B. Systolic Blood Pressure values of control group. C. Diastolic Blood Pressure values of Pb exposure group. D. Diastolic Blood Pressure values of control group. The S1 Data is in the file of S1 Data.xls
(XLS)

S2 Data The value of caspase-3 activity of cardiac and vascular tissue (fold of control). A. Caspase-3 activity in vascular tissue (fold of control). B. Caspase-3 activity in cardiac tissue (fold of control). The S2 Data is in the file of S2 Data.xls
(XLS)

S1 Table Blood lead in the two groups at day 12 and 40 after Pb exposure. The S1 Table is in the file of S1 Table.doc
(DOC)
