# Supplementary material for: Lead Induces Apoptosis and Histone Hyperacetylation in Rat Cardiovascular Tissues
Source: PLoS One. 2015 Jun 15;10(6):e0129091. doi: 10.1371/journal.pone.0129091 (PMC4468051; doi:10.1371/journal.pone.0129091)
Supplement: S1 Table — The S1 Table is in the file of S1 Table.doc (DOC) [file pone.0129091.s003.doc]

**S1 Table. Blood lead in the two groups at day 12 and 40 after Pb exposure.**

| Group | n | control group | Pb exposure group | P |
| --- | --- | --- | --- | --- |
| 12-day | 15 | 68.2±11.1 | 193.3±22.1* | P < 0.05 |
| 40-day | 15 | 69.1 ±12.3 | 245.9±17.9* | P < 0.05 |

*P < 0.05
